# LncRNA SNHG17 regulates cell proliferation and invasion by targeting miR-338-3p/SOX4 axis in esophageal squamous cell carcinoma

**DOI:** 10.1038/s41419-021-04093-w

**Published:** 2021-08-24

**Authors:** Wenhu Chen, Lifang Wang, Xiaoyan Li, Changan Zhao, Liang Shi, Hongguang Zhao, Chen Huang

**Affiliations:** 1 Department of Cell Biology and Genetics, School of Basic Medical Sciences, Xi’an Jiaotong University Health Science Center, Xi’an, China; 2 School of Basic Medical Sciences & Forensic Medicine, Hangzhou Medical College, Hangzhou, China; 3 Department of Pathology, School of Basic Medical Sciences, Xi’an Jiaotong University Health Science Center, Xi’an, China; 4 Department of Ward Pharmacy, Zhejiang Cancer Hospital, Hangzhou, China; 5 Department of Thoracic surgery, Zhejiang Cancer Hospital, Hangzhou, China; 6 Key Laboratory of Environment and Genes Related to Diseases, Xi’an Jiaotong University Health Science Center, Xi’an, China

**Keywords:** Non-coding RNAs, Oesophageal cancer, Oncogenesis

## Abstract

Small nucleolar RNA host gene 17 (SNHG17), a novel functional long noncoding RNA, has been demonstrated to play an essential role in the oncogenesis of several tumors. However, for esophageal squamous cell carcinoma (ESCC) the expression pattern and detailed function of SNHG17 are largely unknown. Hence, we conducted this study to explore potential roles and underlying oncogenic mechanisms for SNHG17 in ESCC progression. Results demonstrated SNHG17 to be markedly upregulated in ESCC. Knockdown of SNHG17 significantly suppressed ESCC cell proliferation, invasion, and epithelial–mesenchymal transition in vitro and tumor growth in vivo. Online database software analysis found miR-338-3p to interact with SNHG17 with the level of miR-338-3p negatively correlated with SNHG17 levels in ESCC samples. Further, miR-338-3p was found to directly target SRY-box transcription factor 4 (SOX4) in ESCC cells. Mechanistic analysis suggested that SNHG17 acts as an endogenous “sponge” competing with miR-338-3p to regulate SOX4, thereby promoting tumor progression. These results suggest that these molecular interactions may be potential therapeutic targets for ESCC.

## Introduction

Worldwide, esophageal cancer (EC) is one of the most prevalent types of malignancy [1]. Esophageal squamous cell carcinoma (ESCC) accounts for ~90% of all esophageal cancers in China and is characterized by high malignancy, multiple metastases, and frequent recurrence, with luminal narrowing and dysphagia [2, 3]. Even with recent treatment advances, including surgical operation, chemotherapy, radiotherapy, and molecular targeted therapy, the 5-year patient survival rate only approximates 30% [4, 5]. Therefore, it is important to understand the underlying mechanisms and molecular pathways that result in tumorigenesis and progression of ESCC. In this manner, it will be possible to develop more effective and precise treatments for this malignancy.

Long noncoding RNA (lncRNA), defined as a form of noncoding RNA greater than 200 nt in length, plays an essential role in mediating cellular processes [6]. Accumulating evidence has shown lncRNAs to be associated with many pathophysiological processes, including tumorigenesis and cancer progression [7–9]. lncRNAs directly or indirectly interact with target RNAs, thereby affecting the generation of RNA [10, 11]. For example, upregulation of lncRNA-MUF in liver cancer contributes to tumor progression by regulating Wnt/β-catenin signaling [12]. The mechanism of action for lncRNAs has been described in a novel post-transcriptional regulation model. In this model lncRNAs competitively sponge microRNAs and shield their target mRNAs, acting as competitive endogenous RNAs (ceRNAs) that neutralize their targets [13, 14]. Another lncRNA, MIR31HG, is highly expressed in pancreatic ductal adenocarcinoma, affecting cell proliferation and invasion by directly interacting with miR-193b [15]. Even though many lncRNAs have been confirmed involvement of tumorigenesis and progression, their biological role and potential mechanism of action in ESCC are not known.

Small nucleolar RNA host gene 17 (SNHG17), a newly identified functional lncRNA, is located on 20q11.23, with a length of 1186 bp (base pairs). It belongs to a large family of noncoding genes known as small nucleolar RNA host genes (SNHGs), which are host genes for small nucleolar RNAs (snoRNAs) [16, 17]. Recent reports have confirmed that SNHG17 is abnormally expressed in tumors and acts as a potential regulator of tumor progression [18–20]. For example, overexpressed SNHG17 was shown to regulate cell proliferation and migration of non-small cell lung cancer (NSCLC) [21]. SNHG17 has been shown to promote gastric cancer progression by downregulation of p15 and p57 [22]. Further, SNHG17 influences melanoma cell proliferation and migration through PI3K-AKT signaling [23]. However, for ESCC the biological function and regulatory mechanisms of SNHG17 are largely unknown.

In our study, we confirmed that SNHG17 was upregulated in tumor tissues and ESCC cells. Moreover, we explored the regulatory effect of SNHG17 on the malignant phenotype of ESCC cell*s* in vitro and in vivo. Our findings suggest that SNHG17 is involved in cell proliferation and invasion of ESCC by regulation of the miR-338-3p/SOX4 axis.

## Results

### lncRNA SNHG17 is overexpressed in ESCC tissues and cells

**Fig. 1:**
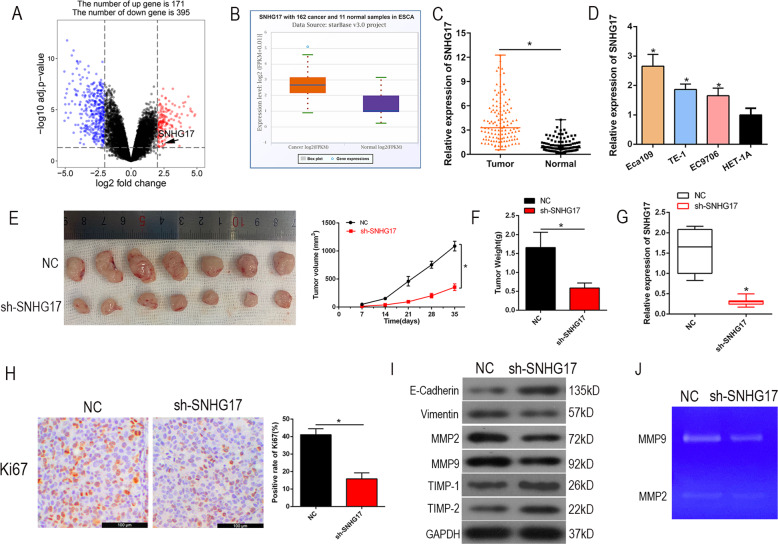
SNHG17 is overexpressed in ESCC tissues and cell lines and SNHG17 knockdown suppresses tumor growth of ESCC in vivo. **A**, **B** The results from the GEO database and starBase showed that SNHG17 expression was obviously upregulated in ESCC tissues compared with normal tissues. The fold change (FC) of genes was assessed by log transformation. |logFC| > 2 and adjusted *P* < 0.05 were defined as the screened threshold. **C**, **D** Expression levels of SNHG17 in clinical samples and cell lines were detected by RT-qPCR. **E**, **F** The tumor volume, weight, and morphology were present. **G** The levels of SNHG17 in xenograft tissues were measured by RT-qPCR. **H** Immunostaining of Ki-67 in xenograft tissues was performed derived from sh-SNHG17 transfected Eca109 cells. **I** EMT-related key proteins E-cadherin, Vimentin, MMP2, MMP9, TIMP-1 and TIMP-2 were detected by Western blot after knockdown of SNHG17 compared with control. **J** Gelatin zymography of activated MMP9 and MMP2 in NC and sh-SNHG17 xenograft tissues. *P < 0.05 vs. control group.

To investigate the effect of SNHG17 in ESCC, we analyzed high throughput sequencing data that compared the expression of SNHG17 in seven pairs of tissues from the GEO data set (GSE111011) and found SNHG17 expression to be upregulated in ESCC samples (Fig. 1A). Next, we assessed the expression of SNHG17 in ESCC based on TCGA data from the StarBase database and found SNHG17 to be upregulated in ESCC tissues compared to normal esophageal tissues (Fig. 1B). Next, ESCC tissues and matched adjacent normal tissues (126 pairs) were collected and analyzed by RT-qPCR. SNHG17 was found to be remarkably overexpressed in ESCC tissues compared to adjacent normal tissues (*P* < 0.05, Fig. 1C). To investigate the clinical significance of SNHG17 in ESCC, we divided ESCC patients into high (*n* = 63) and low (*n* = 63) expression groups based on the median expression level of SNHG17. Correlation analysis for ESCC clinical features and SNHG17 expression showed the grade of ESCC patients to be associated with SNHG17 expression, suggesting that SNHG17 may serve as a prognostic biomarker (*P* < 0.05, Table 1). Further, SNHG17 expression was higher in three ESCC cell lines (Eca109, TE-1, and EC9706) than in the normal esophageal epithelial cell line (HET-1A) (*P* < 0.05, Fig. 1D). These findings suggest SNHG17 to be dysregulated in ESCC and that overexpression may be involved in the progression of the disease.

**Table 1:** Correlations between SNHG17 expression and clinicopathologic features of ESCC patients.

	Expression of SNHG17		
Characteristics	Low	High	*P*-value
Age			0.1527
<60	38	30	
≥60	25	33	
Gender			0.1439
Female	6	2	
Male	57	61	
Grade			0.0289*
G1 + G2	31	19	
G3	32	44	
Pathologic-stage			0.2850
I + II	35	29	
III + IV	28	34	
Pathologic-T			0.5573
T1 + T2	17	20	
T3 + T4	46	43	
Pathologic-N			0.3691
N0	30	25	
N1	33	38	
Pathologic-M			0.6481
M0	61	60	
M1	2	3	

*T* tumor status, *N* regional lymph nodes status, *M* metastasis status, *ESCC* esophageal squamous cell carcinoma.

*P < 0.05 by the chi-square test.

### SNHG17 knockdown suppresses tumor growth of ESCC in vivo

To assess the in vivo biological role of SNHG17 in ESCC tumorigenesis, we inoculated nude mice with Eca109 cells that were stably transfected with sh-SNHG17. Results for tumor morphology, growth curves, and weight showed that SNHG17 knockdown significantly reduced tumor growth in mice (*P* < 0.05, Fig. 1E, F). Tumor tissues from these animals were harvested for RT-qPCR analysis of SNHG17. Reduced expression of SNHG17 was found in tumor tissues derived from sh-SNHG17 stably transfected Eca109 cells compared to controls (*P* < 0.05, Fig. 1G). Ki-67 levels in subcutaneous tumors formed by SNHG17 knockdown in Eca109 cells, as judged by immunohistochemistry (IHC), were less than those of controls (*P* < 0.05, Fig. 1H). Further, suppression of SNHG17 altered the expression of epithelial–mesenchymal transition (EMT)-associated proteins, with high expression of E-cadherin and low expression of vimentin, MMP2, and MMP9 (Fig. 1I). MMP activity was assessed by gelatin zymography. As shown in Fig. 1J, MMP2/9 activity was decreased in sh-SNHG17 treated animals. Collectively, these data suggest that SNHG17 knockdown impairs ESCC tumor growth in vivo.

### Depletion of SNHG17 inhibits ESCC cell proliferation and invasion

**Fig. 2:**
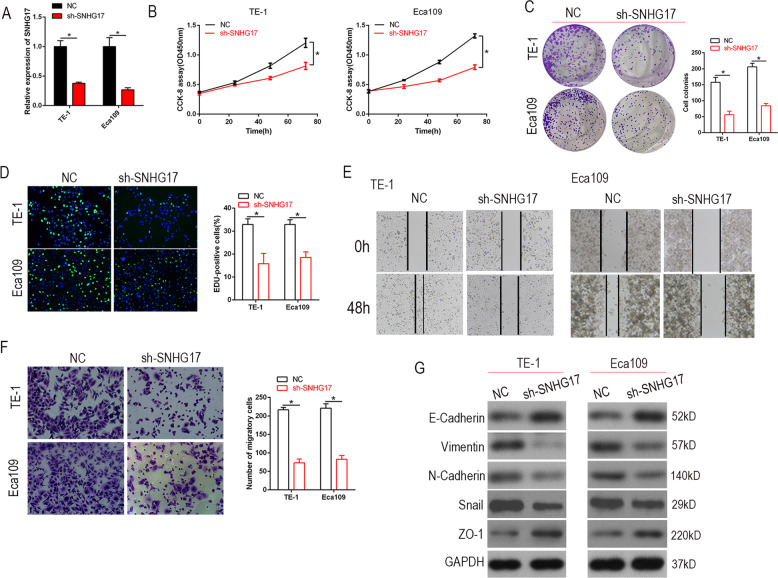
SNHG17 knockdown suppresses ESCC cell proliferation and migration and EMT in vitro. **A** The expression of SNHG17 was knockdown by shRNA in both Eca109 and TE-1 cells. *P < 0.05 vs. NC. **B**, **C** CCK-8 and colony formation assays indicated that SNHG17 knockdown inhibited ESCC cell proliferation. *P < 0.05 vs. NC. **D** EdU immunostaining assays displayed that cell proliferation was restrained by sh-SNHG17 in Eca109 and TE-1 cells. *P < 0.05 vs. NC. **E**, **F** Wound healing and transwell assays were performed to detect the effect of sh-SNHG17 on cell-migration ability. *P < 0.05 vs. NC. **G** Western blot assay revealed that depletion of SNHG17 could reverse the EMT phenotype of Eca109 and TE-1 cells.

SNHG17 was silenced in Eca109 and TE-1 cells with sh-SNHG17. The RT-qPCR analysis demonstrated satisfactory downregulation (*P* < 0.05, Fig. 2A). SNHG17 downregulation significantly suppressed the proliferation of Eca109 and TE-1 cells, as judged by the CCK-8 assay (*P* < 0.05, Fig. 2B). Colony formation and ethynyl deoxyuridine (EdU) incorporation assays also demonstrated SNHG17 knockdown to inhibit the growth of Eca109 and TE-1 cells (*P* < 0 .05, Fig. 2C, D). Likewise, wound healing and transwell assays showed that knockdown of SNHG17 decreased cell invasion by Eca109 and TE-1 (*P* < 0.05, Fig. 2E, F). Further, the western blot demonstrated depletion of SNHG17 to change the expression of EMT phenotypic markers (Fig. 2G). Thus, knockdown of SNHG17 inhibited the proliferation and cell invasion of ESCC cells in vitro.

### SNHG17 acts as a molecular sponge for miR-338-3p in ESCC

**Fig. 3:**
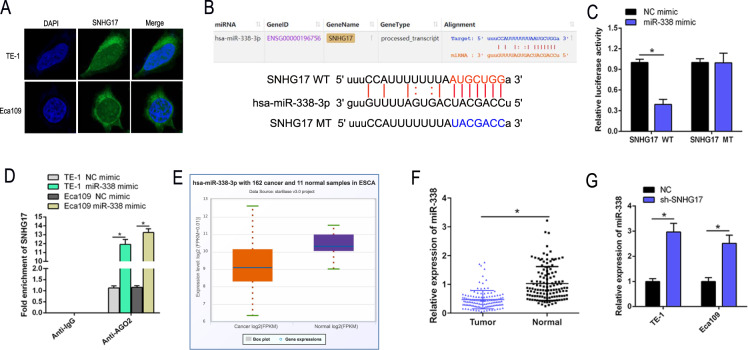
SNHG17 could act as a ceRNA through binding to miR-338-3p. **A** RNA FISH assay of the cellular localization of SNHG17 (labeled in green) in TE-1 and Eca109 cells. **B** The bioinformatics analysis of miRNA database of SNHG17. **C** Relative luciferase activity is decreased in cells transfected with pGL3‐SNHG17-WT and miR-338 mimic than in cells transfected with SNHG17-MT and miR-338 mimic, indicating that miR-338-3p directly binds to SNHG17. *P < 0.05 vs. NC. **D** Interaction between SNHG17 and miR-338-3p was detected by RIP assay. **E** The data from starBase showed that miR-338-3p expression was downregulated in ESCC tissues compared to normal tissues. **F** The miR-338-3p expression was determined by RT-qPCR in clinical samples. *P < 0.05 vs. control group. **G** The RT-qPCR results showed that miR-338-3p expression level was increased by sh-SNHG17 in ESCC cells. *P < 0.05 vs. NC.

The biological function of lncRNAs relates to subcellular location. Fluorescent in situ hybridization (FISH) demonstrated SNHG17 to be primarily located in the cytoplasm of TE-1 and Eca109 cells (Fig. 3A). To clarify whether SNHG17 regulating ESCC progression as a ceRNA, we utilized StarBase V3.0 software to predict the SNHG17 sequence that binds miR-338-3p (Fig. 3B). To confirm this assumption, a dual-luciferase reporter assay was performed in Eca109 cells. As expected, the miR-338 mimics significantly reduced the luciferase activity of SNHG17-WT. However, upregulation of miR-338-3p had no effect on the luciferase activity of SNHG17-MT (Fig. 3C). Further, radio-immuno-precipitation (RIP) assay demonstrated the miR-338-3p mimic to strengthen enrichment of SNHG17 in AGO-2 RIP, whereas its efficacy declined rapidly in response to IgG RIP (Fig. 3D). The StarBase V3.0 database revealed that miR-338-3p was poorly expressed in ESCC samples (Fig. 3E). Expression of miR-338-3p in the 126 pairs of ESCC tissue and matched adjacent normal tissue found downregulation of miR-338-3p in ESCC tumors compared to normal tissue (*P* < 0.05, Fig. 3F). Moreover, RT-qPCR showed that miR-338-3p was upregulated by SNHG17 knockdown in Eca109 and TE-1 cells (*P* < 0.05, Fig. 3G). In summary, SNHG17 appears to function as a molecular sponge for miR-338-3p, inhibiting miR-338-3p function in ESCC.

### miR-338-3p upregulation inhibits proliferation and invasion by Eca109 and TE-1 cells

**Fig. 4:**
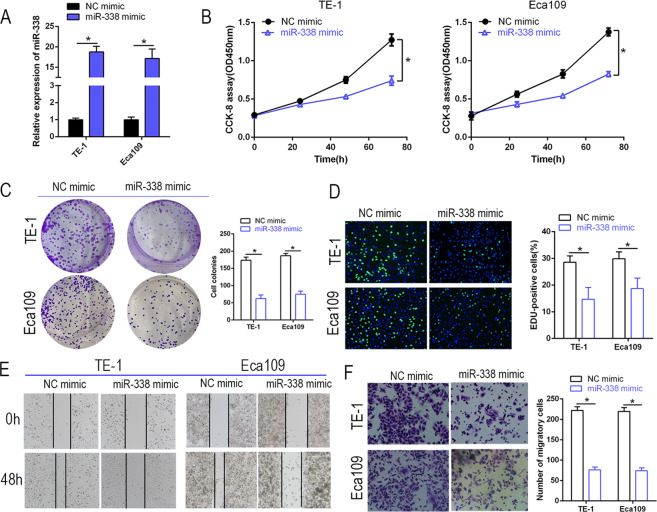
miR-338-3p negatively regulated cell proliferation and invasion in ESCC cells. **A** RT-qPCR was performed to detect the transfection efficiency of miR-338 mimics. **B**, **C** CCK-8 and colony formation assays were performed to determine the proliferation ability of ESCC cells transfected with miR-338 mimics. *P < 0.05 vs. NC. **D** EdU analysis displayed that cell proliferation was restrained by miR-338-3p in ESCC cells. *P < 0.05 vs. NC. **E**, **F** Wound healing and transwell assays were performed to detect the effect of miR-338-3p on cell invasion ability. *P < 0.05 vs. NC.

To reveal the biological effect of miR-338-3p in ESCC, miR-338 and NC mimics were transfected into Eca109 and TE-1 cells. Satisfactory transfection efficiency was obtained by 48 h (Fig. 4A). miR-338-3p upregulation suppressed proliferation of Eca109 and TE-1 cells as judged by CCK-8 assay (*P* < 0.05, Fig. 4B). Colony formation and EdU assays also found reduced proliferative capacity when ESCC cells were transfected with the miR-338 mimic (Fig. 4C, D). Furthermore, wound healing and transwell assays demonstrated weakened invasive capacity for Eca109 and TE-1 cells transfected with the miR-338 mimic (Fig. 4E, F). These results suggest the involvement of miR-338-3p in ESCC progression.

### Restoration of miR-338 expression abolishes the tumor-suppressive effects of SNHG17 knockdown in ESCC cells

**Fig. 5:**
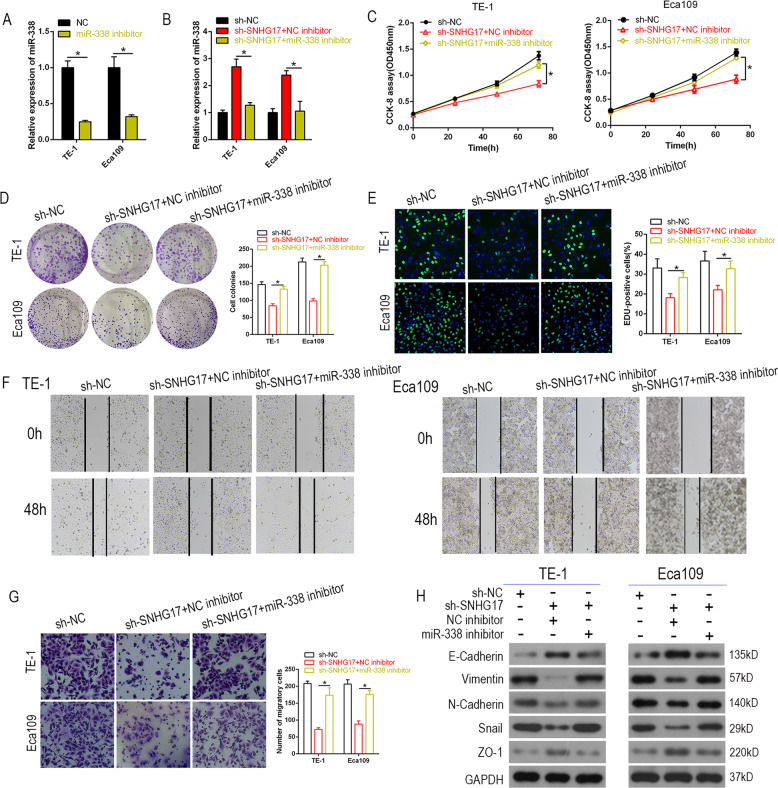
The oncogenic function of SNHG17 in ESCC cells was dependent on miR-338-3p. **A**, **B** The level of miR-338-3p was measured by RT-qPCR in ESCC cells co-transfected with sh-SNHG17 and miR-338 inhibitor. **C**–**E** CCK-8, colony formation assays, and EdU analysis were performed to determine the proliferation ability of ESCC cells co-transfected with sh-SNHG17 and miR-338 inhibitor, respectively. **F**–**H** Wound healing and transwell assays and western blot showed the invasion ability and EMT phenotype in ESCC cells co-transfected with sh-SNHG17 and miR-338 inhibitor. *P < 0.05 vs. NC.

To verify the relationship between SNHG17 and miR-338-3p in ESCC, rescue experiments were conducted to assess the dependency of SNHG17 on miR-338-3p for effects on ESCC proliferation and invasion. miR-338 NC inhibitors were transfected into Eca109 and TE-1 cells in which SNHG17 was knocked down (Fig. 5A, B). CCK-8, colony formation, and EdU assays demonstrated the miR-338 inhibitor to partially reverse the inhibitory effect on cell proliferation induced by sh-SNHG17 in Eca109 and TE-1 cells (Fig. 5C–E). Likewise, ESCC wound healing, transwell assays, and western blot demonstrated weakened invasive capacity with SNHG17 knockdown, which was in part abrogated by the miR-338 inhibitor (Fig. 5F–H). Taken together, these findings suggest that SNHG17 contributes to the progression of ESCC through targeting of miR-338-3p.

### miR-338-3p directly targets SOX4 mRNA in ESCC cells

**Fig. 6:**
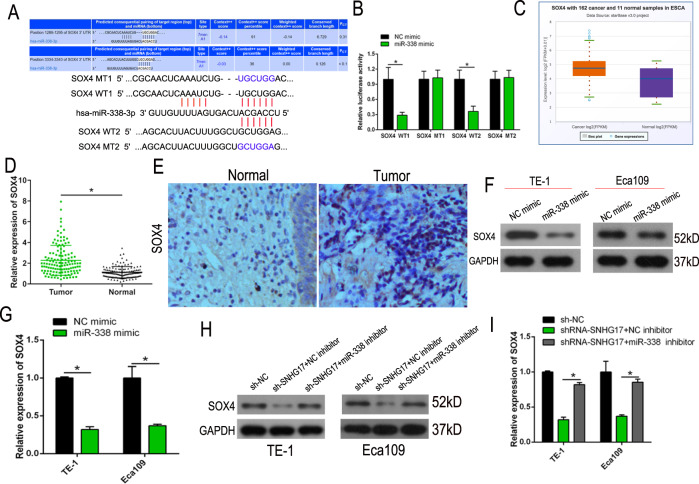
miR-338-3p directly targets SOX4 mRNA in ESCC cells. **A** The bioinformatics analysis of miRNA database of miR-338-3p. **B** Relative luciferase activity is decreased in cells transfected with pGL3‐SOX4-WT1/2 and miR-338 mimic than in cells transfected with SOX4-MT1/2 and miR-338 mimic. *P < 0.05 vs. NC. **C** The data from starBase showed that SOX4 expression was upregulated in ESCC tissues compared to normal tissues. **D**, **E** The SOX4 expression was detected by RT-qPCR and IHC in clinical samples. *P < 0.05 vs. control group. **F**, **G** The RT-qPCR and western blot results showed that SOX4 expression level was increased by a miR-338 mimic in ESCC cells. **H**, **I** SNHG17 downregulation reduced the level of SOX4, and this was reversed by miR-338 inhibitor in cells. *P < 0.05 vs. NC.

To explore the molecular role of miR-338-3p in ESCC progression, potential targets of miR-338-3p were predicted with StarBase V3.0 software. Two feasible, highly conserved binding sites were found in the 3′ UTR of SOX4 mRNA (Fig. 6A). Luciferase activity was markedly lower in SOX4-WT1/2 transfected cells that were co-transfected with the miR-338 mimic. However, no inhibition of luciferase activity was observed for cells that were transfected with SOX4-MT1/2 (*P* < 0.05, Fig. 6B). StarBase V3.0 database analysis found SOX4 to be overexpressed in ESCC samples compared to adjacent normal tissue (Fig. 6C). Consistent with this finding, RT-qPCR and IHC demonstrated SOX4 to be more highly expressed in the 126 ESCC tissue than in matched tissue (*P* < 0.05, Fig. 6D, E). Furthermore, RT-qPCR and western blot demonstrated SOX4 expression to be noticeably suppressed by miR-338-3p in both Eca109 and TE-1 cells (*P* < 0.05, Fig. 6F, G). Rescue experiments showed that SNHG17 knockdown significantly reduced the expression level of SOX4, whereas a miR-338 inhibitor reversed the negative effect of SNHG17 knockdown in comparison to the negative control (Fig. 6H, I). These results demonstrate the negative relationship between miR-338-3p with SOX4 expression in ESCC cells.

### Restoration of SOX4 reverses the effect of miR-338-3p on ESCC cell proliferation and invasion

**Fig. 7:**
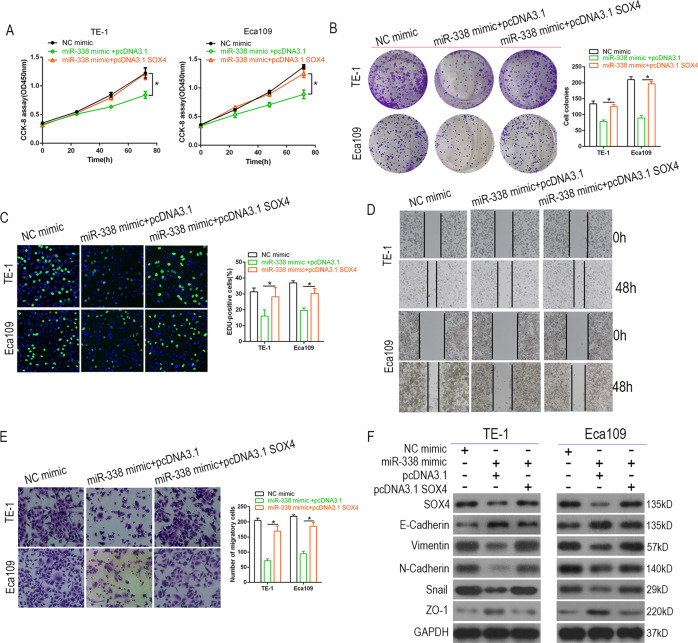
Restoration of SOX4 reverses the effects of miR-338-3p on ESCC cells proliferation, invasion, and EMT process. **A**–**C** CCK-8, colony formation assays, and EdU analysis were performed to determine the proliferation ability of ESCC cells co-transfected with miR-338 mimic and SOX4, respectively. **D**–**F** Wound healing and transwell assays and western blot showed the invasion ability and EMT phenotype in ESCC cells co-transfected with miR-338 mimic and SOX4. *P < 0.05 vs. NC.

Based on the above, SOX4 may be involved in SNGH17/miR-338-3p-mediated tumor progression. We performed CCK-8, colony formation, and EdU assays and found significant reductions in the proliferative capacity of cells transfected with miR-338-3p. This effect was reversed by upregulation of SOX4 (*P* < 0.05, Fig. 7A–C). Wound healing, transwell assays, and western blot showed miR-338-3p to inhibit EMT and cell invasiveness, which were blocked by overexpression of SOX4 (Fig. 7D–F). In summary, all data displayed that SNHG17 facilitates cellular multiplication and invasion in vitro by regulation of the miR-338-3p/SOX4 axis.

## Discussion

lncRNAs play a vital role in cell physiology, pathology, the regulation of tumorigenesis, and cancer progression [7, 24]. Zhang et al. found the lncRNA, HOXC-AS3, to be overexpressed in gastric cancer and to mediate oncogenic gene transcriptional regulation [25]. lncRNA, MALAT1, is a suppressor of breast cancer tumor metastasis by binding and inactivating TEAD [26]. However, potential mechanisms of action for ESCC lncRNAs are not known. Hence, we analyzed high throughput sequencing GEO data for ESCC associated lncRNAs. Interestingly, results showed that SNHG17, a newly discovered lncRNA, is significantly upregulated in ESCC tissue compared to adjacent normal tissue. Correlation analysis of clinical characteristics and SNHG17 expression in ESCC showed that ESCC patient Grade was associated with SNHG17 expression. In this study, overexpression of SNHG17 was found in ESCC tissues and cell lines. Further, SNHG17 was shown to contribute to the regulation of ESCC cellular proliferation and invasion via the miR-338-3p/SOX4 axis. Results of this study demonstrate SNHG17 to be involved in the development of ESCC. Hence, SNHG17 may be a potential therapeutic target for ESCC.

Recent studies suggest that abnormal expression of SNHG17 is associated with several tumors, including gastric cancer, non-small-cell lung cancer, colorectal cancer, melanoma, breast cancer, glioma, and tongue squamous cell carcinoma [18, 19, 21–23]. SNHG17 plays a prominent role in promoting the progression of these tumors. Nevertheless, the function of SNHG17 in ESCC is unknown. Herein, loss-of-function assays demonstrated SNHG17 silencing to suppress ESCC cell proliferation, colony formation, EMT progression, and invasion in vitro. Likewise, silencing SNHG17 inhibited tumor growth in vivo. These results suggest that SNHG17 may have an oncogenic role in ESCC progression. However, the mechanism of action for SNHG17 in ESCC requires further exploration.

In a current lncRNA regulation model, lncRNAs competitively sponge miRNAs and shield their target mRNAs, achieving downstream target regulation by acting as ceRNAs [14, 27]. Using bioinformatics analysis, we predicted that SNHG17 is a possible target for miR-338-3p. miR-338-3p is known to act as a tumor suppressor gene in thyroid cancer, squamous cell carcinoma, hepatocellular carcinoma, gastric cancer, and prostate cancer [28–32]. For example, miR-338-3p has been found to inhibit thyroid cancer cell growth through direct targeting of AKT3 [28]. Further, miR-338-3p regulates EMT formation by targeting ZEB2 and MACC1/Met/Akt pathways in gastric cancer [30]. Our experiments verified the downregulation of miR-338-3p in ESCC tissues. Luciferase activity and RT-qPCR assays identified a negative relationship between SNHG17 and miR-338-3p in ESCC. Consistent with the effect of SNHG17 knockdown, a miR-338 mimic inhibited cell proliferation, colony formation, EMT progression, and invasion by ESCC cells. Therefore, SNHG17 may be a molecular sponge of miR-338-3p during ESCC progression.

To further assess the function of SNHG17 in tumorigenesis and tumor progression, we analyzed a downstream target of miR-338-3p, SOX4, and predicted two binding sites within the 3′UTR of SOX4. SOX4 belongs to the SOX transcription factor family, which binds the DNA minor groove. This binding changes the structure of chromatin, promoting the formation of transcriptional enhancer complexes. Previously, an oncogenic role for SOX4 was identified in liver cancer, acute myeloid leukemia, and pancreatic cancer [33–36]. The results herein demonstrate SOX4 to be overexpressed in ESCC and downregulated by miR-338 mimics in ESCC cell lines. Rescue experiments demonstrated that upregulation of SOX4 reversed the effect of miR-338 mimics on ESCC cell proliferation, invasion, and EMT phenotype. These results demonstrate the importance of SNHG17/miR-338-3p regulation of SOX4 in ESCC progression.

In summary, we discovered that lncRNA SNHG17 is upregulated in ESCC tissues and cells. The impact of SNHG17 on cell proliferation, invasion, and EMT progression demonstrates a role for SNHG17 in ESCC oncogenesis. With regard to the mechanism of action, we demonstrated SNHG17 to directly target miR-338-3p and suppress miR-338-3p by acting as a molecular sponge for miR-338-3p. Thus, we propose that SNHG17 regulates ESCC cell proliferation and invasion by targeting the miR-338-3p/SOX4 axis. These results may provide the foundation upon which to build novel therapeutic treatments for ESCC.

## Materials and methods

### Clinical specimens and cell culture

A total of 126 pairs of ESCC tissue and adjacent normal tissue were acquired from the Thoracic Surgery Department of Zhejiang Cancer Hospital. The tissues were immediately snap-frozen in liquid nitrogen until RNA extraction. None of the ESCC patients received any adjuvant therapy prior to surgery. This study was approved by the Medical Ethics Committee of the University of Zhejiang Cancer Hospital and all patients provided written informed consents. The ESCC cell lines (Eca109, TE-1, and EC9706) and the human esophageal epithelial cell line (HET-1A) were obtained from the American Type Culture Collection (ATCC). All cells were cultured in RPMI 1640 medium supplemented with 10% fetal bovine serum (Gibco), 100 μg/mL streptomycin (Invitrogen), and 100 U/mL penicillin (Invitrogen) in a humidified incubator containing 5% CO_2_ at 37 °C.

### Immunohistochemistry

Immunohistochemistry (IHC) was performed on 5-μm-thick FFPE tumor tissue sections. Slides were stained with primary antibodies reactive with SOX4. Then, washed and incubated with horseradish peroxidase-conjugated secondary antibodies. Immuno-peroxidase staining was developed using the DAB system according to the manufacturer’s instructions. Slides were counterstained with hematoxylin, dehydrated, and coverslipped using a mounting solution. IHC signals were enumerated in seven random 20× fields, with signals averaged for each slide.

### Plasmids, oligonucleotides, and transfection

The short-hairpin SNHG17 (sh-SNHG17: 5′-GAUUGUCAGCUGACCUCUGUCCUGU-3′) and negative control shRNA (sh-NC: 5′-UUCUCCGUUCGUGUCACGUUU-3′) were designed and constructed by GenePharma Co., Ltd. (Shanghai, China). The miR-338-3p mimic (5′-UUUGAGCAGCACUCAUUUUUGC-3′), NC mimic (5′-CAGUACUUUUAGUGUGUACAA-3′), miR-338 inhibitor (5′-CAACAAAAUCACUGAUGCUGGA-3′), and NC inhibitor (5′-CAGUACUUUGUGUAGUACAA-3′) were purchased from RiboBio Co., Ltd. (Guangzhou, China). The full-length cDNA of the SOX4 gene was PCR-amplified and then inserted into the pcDNA3.1 vector. Oligonucleotides and plasmids were transfected into cells using Lipofectamine 2000 (Invitrogen) following the manufacturer’s protocol.

### RT-qPCR assay

Total RNA was extracted from ESCC tissues and cells utilizing TRIzol reagent (Invitrogen). cDNA synthesis was with a PrimeScript RT reagent Kit (Takara, Japan). RT-qPCR was performed with SsoFast EvaGreen Supermix (Bio-Rad, USA) and an ABI Prism 7500 Sequence Detection System according to the manufacturer’s instructions. miR-338-3p was detected with a Mir-X miRNA First-Strand Synthesis Kit (Clontech, USA) according to the manufacturer’s instructions. Primer sequences were as follows: SNHG17 F:GTTCCTGGGGCTTGGATGAT, SNHG17 R: GATCTAAGGCTGAGACCCACG; β-actin F:TGGCACCCAGCACAATGAA, β-actin R: CTAAGTCATAGTCCGCCTAGAAGCA; and miR-338-3p F:TCCAGCATCAGTGATTTTGTTG. Relative gene expression was normalized to β-actin or U6 based on the 2^−ΔΔCt^method.

### Western blot

Total protein was isolated by lysing cells with RIPA buffer (Beyotime, China) and was quantified with a BCA assay kit (Sangon, China). Total protein (50 μg) was separated by electrophoresis (10% SDS-PAGE) and transferred to a PVDF membrane (Millipore, USA). After treatment with a skim milk powder for 2 h at room temperature, the membrane was incubated overnight with primary antibody reactive with either; E-cadherin (14472,CST), Vimentin (5741,CST), N-cadherin (13116,CST), SOX4 (PA5-41442, Thermo Scientific), Snail (3879,CST), ZO-1 (13663,CST), TIMP-1 (8946,CST), TIMP-2 (5738,CST), or GAPDH (5174,CST) at 4 °C. The next day, secondary antibodies conjugated with horseradish peroxidase (HRP) were incubated with the membranes for 2 h. Protein signals were detected with ECL reagents (Sangon, China).

### Gelatin zymography

Gelatin zymography was carried out as described previously [37]. Protein extracts were diluted with a non-reducing loading buffer and loaded onto 8% SDS gels containing 0.2% gelatin. Electrophoresis was performed at 20 mA per gel for 2.5 h. After electrophoresis, gels were soaked for 1 h in 2.5% Triton X-100, followed by incubation for 18 h at 37 °C in zymography buffer. The gels were stained with Coomassie brilliant blue R-250 followed by de-staining.

### CCK-8 assay and EdU analysis

Cell counting Kit-8 (CCK-8) assay was used to assess cell viability. ESCC cells were seeded in 96-well plates at a density of 1 × 10^4^/mL. After incubation for 0, 24, 48, and 72 h, 10 μL of CCK-8 solution (Djingo, Japan) was added to each well and the absorbance at 450 nm was measured using a microplate reader after incubation at 37 °C for 2 h. The experiments were repeated in triplicate, independently.

An ethynyl deoxyuridine (EdU) kit (Invitrogen) was used to assay cell proliferation based on the manufacturer’s instructions. Treated cells were observed by fluorescence photo-microscopy with results quantified by counting at least six random fields

### Colony formation assay

Following transfection for 24 h, ESCC cells were placed into six-well plates and cultured with a complete medium for 2 weeks. The medium was replaced every 3 days. Cell colonies were fixed with paraformaldehyde and stained with 0.1% crystal violet for 20 min. Visible colonies were counted for quantification of results.

### Cell migration and invasion assays

A wound-healing assay was used to assess cell migration. Transfected cells were cultured in six-well plates to full confluence. Cell monolayers were manually scraped to create wound areas using a pipette tip. Progression of migration was observed and photographed at 0 and 48 h after wounding.

Transwell assays were used to evaluate cell invasion. After 24 h of transfection, ESCC cells in FBS-free medium were placed into the upper chambers that had been pre-coated with Matrigel (Corning, USA), whereas the lower chambers contained a medium with 10% FSB. Following 24 h incubation, the invading cells were fixed with paraformaldehyde and stained with 0.1% crystal violet, and enumerated by light microscopy.

### RIP assay

Magna RIP RNA-Binding Protein Immunoprecipitation Kits (Millipore, USA) were used for RIP assessment based on the manufacturer’s instructions. TE-1 and Eca109 cells transfected with mimics were collected and lysed with RIP lysis buffer containing RNasin and protease inhibitors. The lysate was centrifuged at 12,000 × *g* for 30 min followed by a collection of the supernatant, which was incubated with magnetic beads coated with anti-human AGO-2 or anti-IgG for 6 h at 4 °C. The enrichment of SNHG17 was subsequently determined by RT-qPCR.

### Luciferase reporter assay

Sequences of lncRNA SNHG17 and 3′-UTR of SOX4 were amplified and sub-cloned into the pGL3 vector. Potential miR-338-3p binding sites in SNHG17 or SOX4 3′UTR were mutated and inserted into the pGL3 plasmid to generate control groups. These plasmids and the miR-338 mimic and the NC mimic were co-transfected into ESCC cells using Lipofectamine 2000. The Dual-Luciferase Reporter Assay System (Promega, USA) was used to measure and normalize luciferase activity after 24 h of transfection.

### Tumor xenograft

Male BALB/c nude mice (5 weeks old) were purchased from Shanghai SLAC Laboratory Animal Co., Ltd. (Shanghai, China). Eca109 cells stably transfected with sh-NC or sh-SNHG17 were implanted subcutaneously into the flank of mice. Tumor growth was measured every week with a vernier caliper and calculated by using the formula: volume = (length × width^2^)/2. After 5 weeks, all mice were performed euthanasia with anesthesia. Subsequently, tumor xenografts were removed, weighed, and prepared for Ki-67 IHC staining according to the manufacturer’s protocol.

### FISH assay

A lncRNA SNHG17 FISH assay was performed using RNA FISH kits (GenePharma, Shanghai, China) according to the manufacturer’s instructions. FAM-labeled SNHG17 probes were designed and synthesized by GenePharma.

### Statistical analysis

All data were analyzed with GraphPad Prism 7.0 software. A Student’s *t*-test was used to analyze two-group comparisons, and two-way ANOVA was used for multiple group comparisons. The associations among SNHG17 and clinical characteristics of ESCC patients were assessed by the chi-square test. Statistical correlations among miR-338-3p and target sequences were evaluated by Spearman’s analysis. Differences were considered statistically significant at *P* < 0.05.

## Data Availability

The authors declare that all data supporting the findings of this study are available within the article.
